# Calculating optic nerve planning organ at risk volume margins for stereotactic radiosurgery using optic nerve motion determined using MRI

**DOI:** 10.1093/bjr/tqae201

**Published:** 2024-10-04

**Authors:** Sagar Sabharwal, Geoff Heyes, George S J Tudor, Robert Flintham, Swarupsinh Chavda, Paul Sanghera

**Affiliations:** Dept Medical Physics, University Hospitals Birmingham NHS Foundation Trust, Birmingham, B152TH, United Kingdom; Dept Medical Physics, University Hospitals Birmingham NHS Foundation Trust, Birmingham, B152TH, United Kingdom; Dept Physics, University of Birmingham, Birmingham, B152TT, United Kingdom; Dept Medical Physics, University Hospitals Birmingham NHS Foundation Trust, Birmingham, B152TH, United Kingdom; Dept Medical Physics, University Hospitals Birmingham NHS Foundation Trust, Birmingham, B152TH, United Kingdom; Dept Radiology, University Hospitals NHS Foundation Trust, Birmingham, B152TH, United Kingdom; Dept Oncology, University Hospitals NHS Foundation Trust, Birmingham, B152TH, United Kingdom

**Keywords:** optic nerve motion, stereotactic radiosurgery, cyberknife, radiotherapy, PRV margins, MRI

## Abstract

**Objectives:**

The combination of sharp dose gradients in stereotactic radiosurgery (SRS) and minute optic nerve motion may significantly increase dose to the optic nerves when treating perioptic lesions. The aim of this study was to calculate optic nerve planning organ at risk volume (PRV) margins for CyberKnife SRS treatment planning.

**Methods:**

MRI scans were taken of 10 healthy volunteers looking left, right, up, down, and straight ahead to measure optic nerve motion. The measured optic nerve motion and the uncertainties in the technical accuracy of CyberKnife were used to calculate optic nerve PRV margins.

**Results:**

Two optic nerve PRV margins were calculated: a non-isotropic margin of $m_{L/R,\mathrm{PRV}}=3\;\mathrm{mm}$, $m_{\mathrm{Sup}/\mathrm{Inf},\mathrm{PRV}}=2\;\mathrm{mm}$, and $m_{\mathrm{Ant}/\mathrm{Post},\mathrm{PRV}}=1\;\mathrm{mm}$ which considers the full range of motion measured in a worst case scenario; and an isotropic margin of $m_{\mathrm{PRV}}=1\;\mathrm{mm}$ which considers a scenario where patients are asked to look neutrally during imaging and treatment. Applying these PRVs to 8 historical sphenoid wing meningioma CyberKnife plans showed tolerance levels may be exceeded due to optic nerve motion.

**Conclusions:**

Optic nerve PRV margins may be needed in CyberKnife planning to reduce risk to the optic nerves. The use of a $m_{\mathrm{PRV}}=1\;\mathrm{mm}$ PRV to account for organ motion, along with instructing patients to hold their gaze neutrally during imaging and treatment, may be a suitable organ sparing strategy.

**Advances in knowledge:**

Measured optic nerve motion and the technical accuracy of the CyberKnife system have been used to calculate optic nerve PRV margins.

## Introduction

The optic nerves move with a patient’s gaze^1^^,^^2^ even when looking at a fixed point.^3–5^ This presents a challenge in radiotherapy treatment as the position of the optic nerves during treatment delivery may differ from the CT and MRI scans taken for treatment planning. During stereotactic radiosurgery (SRS) high energy photon beams are delivered with high precision over a small number of treatment sessions, with a high dose per fraction (typically 1-5 fractions per course).^6^ The steep dose gradients achievable in SRS techniques mean this treatment technique can be used to treat lesions close to the optic pathways. It is important to manage potential movement of the optic nerves during treatment delivery to prevent unintended exposure. Exposing an optic nerve to a high radiation dose can result in radiation-induced optic neuropathy, a late complication of radiotherapy, causing progressive irreversible vision loss.^7–9^

Radiosensitive organs, such as the optic nerves, are contoured on the planning CT/MRI scans to create organ at risk (OAR) volumes^10^ in order to reduce dose to these organs during treatment planning. OAR volumes can be expanded to account for organ motion and any other uncertainties in radiotherapy treatment to form planning organ at risk volumes (PRVs). Work looking into accounting for optic nerve motion during radiotherapy treatment has been carried out previously, either by deforming contours^11^ or calculating optic nerve PRV margins.^1^^,^^12^ It has been shown that optic nerve motion can result in a non-negligible effect on radiation dose to the optic nerves,^1^^,^^2^^,^^7^^,^^11^^,^^12^ with increases in optic nerve dose of up to 9% in SRS.^2^

The aim of this study was to calculate optic nerve PRV margins for use in SRS which take into account the positional uncertainty of the optic nerves, found by imaging the optic nerve motion of healthy volunteers using MRI, and incorporating the geometric uncertainties in CyberKnife treatment.

### Stereotactic radiosurgery

**Figure 1.**
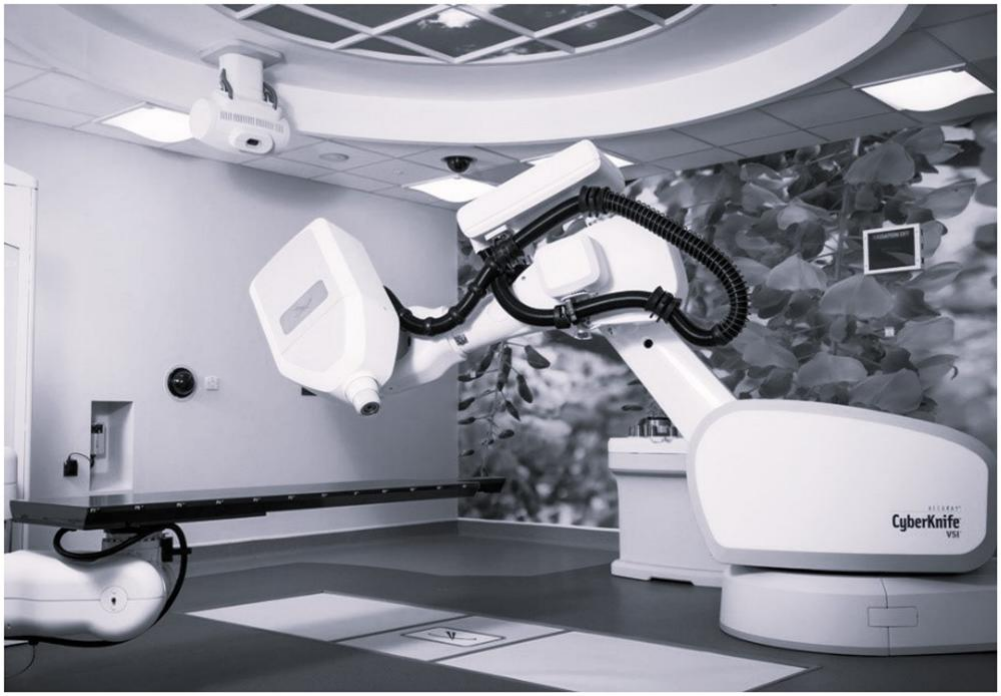
The CyberKnife system at Queen Elizabeth Hospital Birmingham. The linac and couch are each mounted on robotic arms with 6 degrees of freedom. The kV imaging system, consisting of 2 ceiling mounted X-ray tubes and 2 detector panels in the floor, detects patient movement. The CyberKnife system adjusts the position of the linac and couch to account for motion if necessary.

CyberKnife is capable of submillimeter accuracy.^13^ This accuracy is achieved using a linac and couch, each mounted on separate robotic arms with 6 degrees of freedom, and a kV imaging system (Figure 1). This kV imaging system tracks the position of the skull for intracranial treatments and the CyberKnife system adjusts the robotic arms to account for any patient movement detected.^13^^,^^14^ This assumes the position of the treatment target is fixed in relation to the position of the skull. However, the optic nerves can move while the skull is stationary and therefore the position of the optic nerves may change relative to the radiation field during treatment. PRV margins may need to be considered when treating lesions in close proximity to the optic nerves with CyberKnife.

### Radiation-induced optic neuropathy

Radiation-induced optic neuropathy (RION) is a late complication of SRS that can occur following irradiation of the anterior visual pathway, resulting in progressive irreversible vision loss.^7–9^ RION typically occurs within 3 years of radiotherapy treatment^7–9^ but is quite rare, with an occurrence of approximately 1-2% following SRS.^7^ For lesions directly adjacent to the optic pathways, any movement may result in the optic nerves moving into regions of high dose. The risk is compounded by the higher dose per fraction of SRS, and the lower number of fractions compared to standard radiotherapy, as the effective blurring of dose over several deliveries is reduced.

## Methods

A 3 T Siemens Magnetom Skyra MRI scanner with a 32-channel head coil (Siemens Healthineers AG, Erlangen, Germany) was used to image 10 healthy volunteers using a T1 weighted 3D gradient echo volumetric interpolated breath-hold examination (VIBE) sequence (Table1). Repeat scans were taken with 3 of these volunteers.

**Table 1.** Settings used for the T1 VIBE sequence for MRI of the optic nerves using a 3 T Siemens Magnetom Skyra MRI scanner with a 32-channel head coil.

Scan length	3 min 26 s
Voxel size	0.7 × 0.7 × 1.0 mm
Slices	36
TE	1.64 ms
TR	4.83 ms
Phase oversampling	0%
Slice oversampling	22.2%
Field of view read	220 mm
Field of view phase	100%
Averages	3
Flip angle	13.0 deg
Base resolution	320

**Figure 2.**
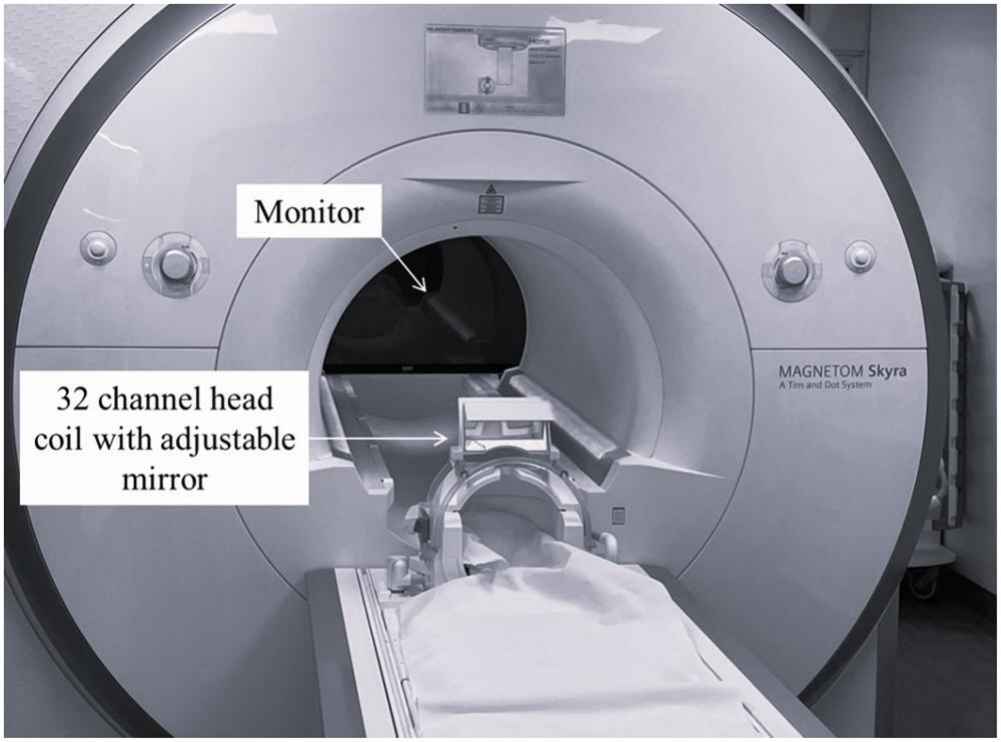
3 T Siemens Magnetom Skyra MRI scanner with a 32-channel head coil.

**Figure 3.**
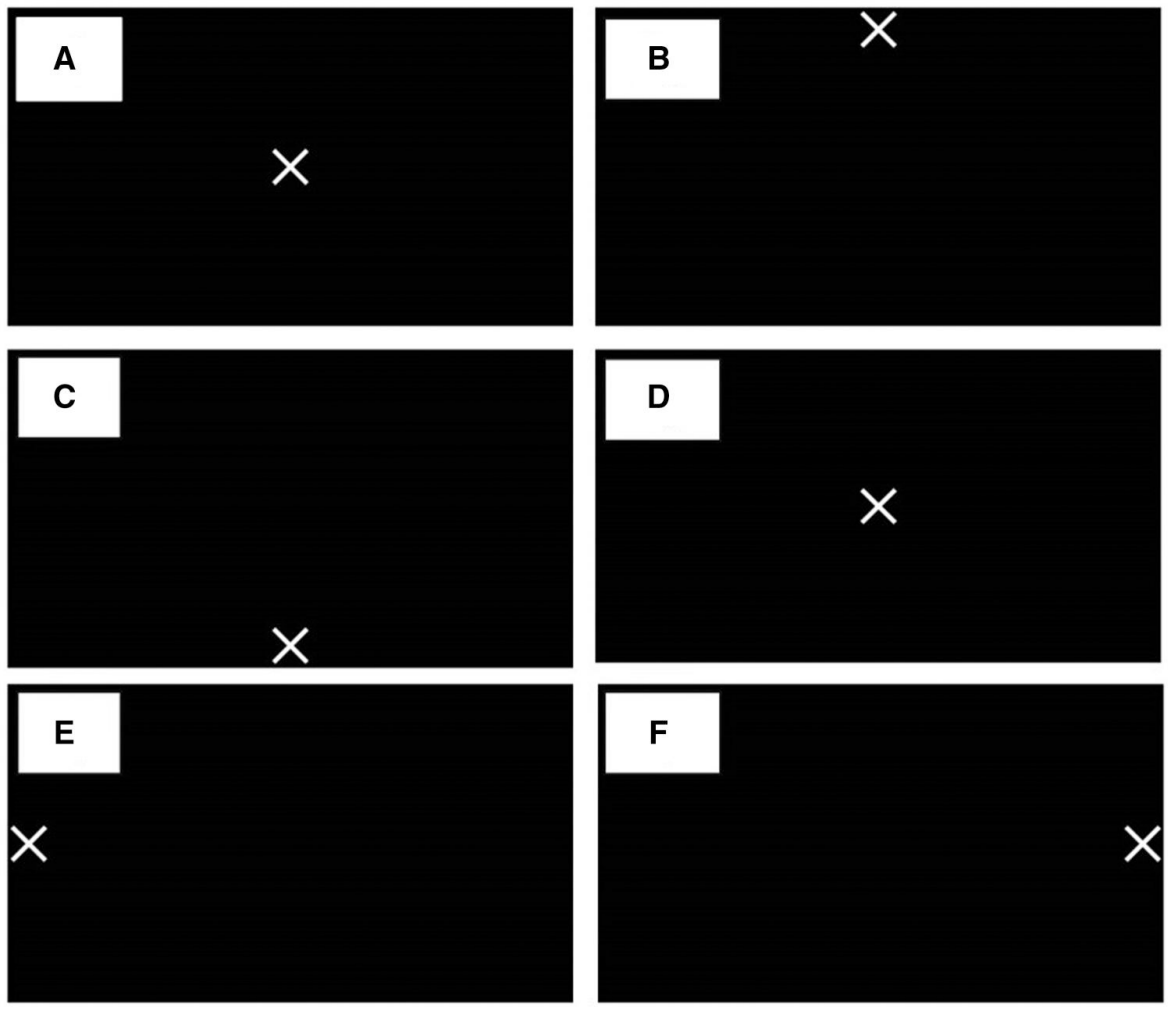
Images shown to the volunteer on the monitor in Figure 2 whilst they were imaged, with the cross in the: (A) neutral, (B) up, (C) down, (D) neutral, (E) left, and (F) right positions.

The eye position protocol used for this project uses the mirror and monitor set-up shown in Figure 2 with the images in Figure 3 displayed on the monitor. First the neutral position in Figure 3 was used to set-up the mirror position so that it is directly over the neutral gaze of the volunteer positioned in the MRI bore. All volunteers were asked to look at each cross to ensure they were visible before scanning. The first scan was taken with the volunteer looking at the neutral position. This was followed by the remainder of the axial scans in the order shown in Figure 3. A 30 s break between scans was given to allow the volunteer to rest their eyes. The aim was to measure non-strenuous eye motion to mimic motion observed during treatment. No volunteer reported any discomfort.

**Figure 4.**
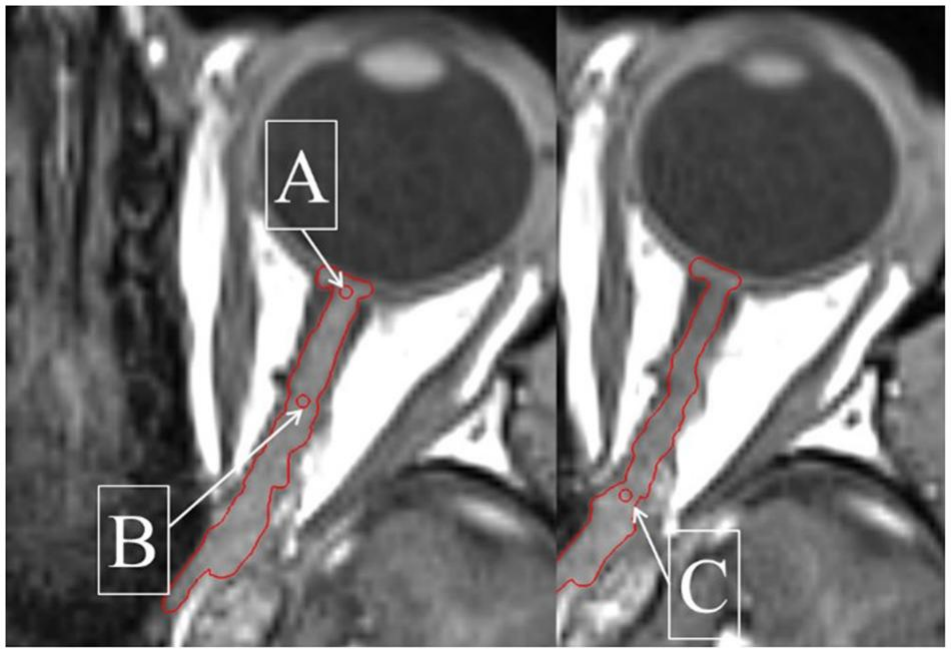
Placement of position markers used to measure optic nerve motion: position A is the point where the optic nerves meet the optic disc, position C is the point where the optic nerves exit the optic canal and position B is the midpoint between positions A and C. Position C is on a different slice to positions A and B here.

All MRI scans acquired in an imaging session were rigidly registered in Precision (Accuray Inc, Sunnyvale, United States) to the first neutral position scan acquired using bony anatomy. Optic nerve position was measured by placing 3 markers on the optic nerves in axial slices along the optic canal in the MRI image using the contouring tools in Precision. Consistency in marker position placement was achieved using proximity to anatomical landmarks. The co-ordinates of each of these markers were recorded for both optic nerves across each gaze scan for each volunteer (see Figure 4).

## Results

### Optic nerve motion

**Figure 5.**
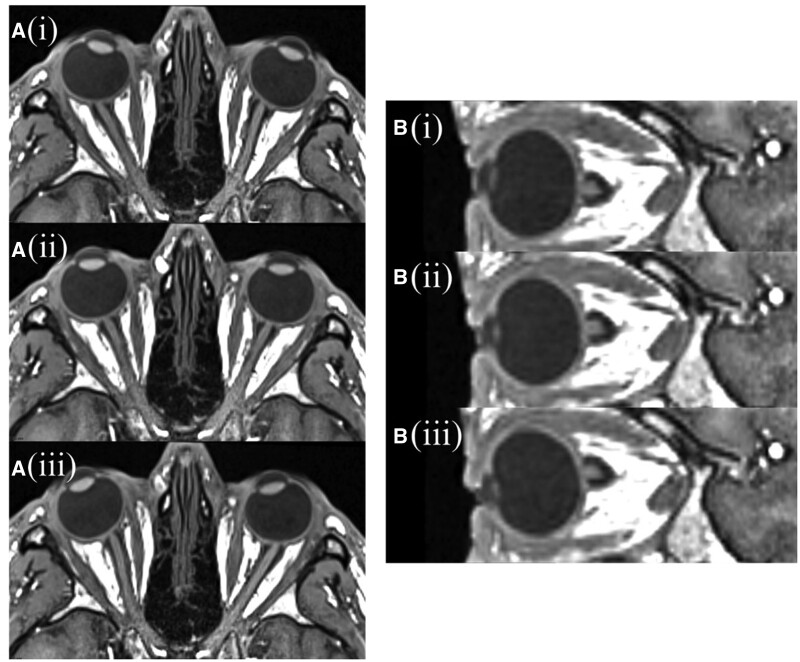
MRI images showing optic nerve motion: (A) axial scans with the volunteer looking (i) left, (ii) neutral, and (iii) right; and (B) coronal scans with the volunteer looking (i) up, (ii) neutral, (iii) down.

Sample images of optic nerve displacement observed are shown in Figure 5. The mean optic nerve displacement magnitude and the corresponding 1 sample standard deviation measured in the lateral and anteroposterior directions at positions A, B, and C are given in Table 2. The greatest optic nerve displacement is seen at position A, at the anterior end of the optic nerves. It was also found there was a mean displacement of position A between the first and second neutral scans from the same scan session of 0.9 ± 0.6 mm.

**Table 2.** Mean optic nerve displacement magnitude measured at positions A, B and C (see Figure 4) along the optic nerve.

Position	Mean displacement magnitude (mm)			
	Left optic nerve		Right optic nerve	
	Lateral	Anteroposterior	Lateral	Anteroposterior
A	5.6 ± 0.8	3.8 ± 0.8	5.7 ± 0.5	3.8 ± 0.7
B	2.9 ± 0.7	2.1 ± 0.7	2.8 ± 0.4	2.0 ± 0.4
C	1.5 ± 0.5	1.2 ± 0.5	1.0 ± 0.5	1.1 ± 0.4

Ten healthy volunteers were imaged and repeat imaging was carried out with 3 out of these 10 volunteers.

The mean lateral and anteroposterior optic nerve displacement magnitude measured at the anterior end of the optic nerves (position A) in the first and repeat scans for volunteers who had repeat scans is given in Table 3. This data shows little variation in magnitude between the first and repeat sessions; measurements were within 1 standard deviation of each other. The mean difference in displacement of both optic nerves at the anterior end of the optic nerves (position A) from the first neutral position in the first session to the first neutral position in the repeat session was found to be 1.3 ± 0.7 mm. This measured motion is within 1 standard deviation of the mean displacement between the first and second neutral scans within the same session (at position A) of 0.9 ± 0.6 mm discussed earlier. This suggests that optic nerve motion may vary to a similar degree inter-fraction, intra-fraction, and between imaging and treatment.

**Table 3.** Lateral and anteroposterior mean optic nerve displacement measured at position A on the optic nerve (see Figure 4) in the first and repeat sessions for the 3 volunteers where repeat sessions were taken.

	Mean displacement magnitude (mm)			
	Left optic nerve		Right optic nerve	
	Lateral	Anteroposterior	Lateral	Anteroposterior
First scan	5.5 ± 1.1	4.1 ± 0.1	5.7 ± 0.8	3.8 ± 0.6
Repeat scan	6.0 ± 0.5	4.2 ± 0.7	5.7 ± 0.6	4.2 ± 0.2

### Planning risk volume

An adapted form of the van Herk model^15^^,^^16^ was used to calculate optic nerve PRV margins:


(1) $$m_{\mathrm{PRV}}=1.3\Sigma \pm 0.5\sigma ,$$

where Σ and σ are the total standard deviations of the systematic and random errors in treatment; 1.3 and 0.5 are dimensionless constants calculated by McKenzie *et al*,^16^ where 1.3 corresponds to a 90% confidence interval the optic nerve will be within the PRV. McKenzie *et al* suggest random errors will have a minimal impact on dose to a PRV in cases where the tolerance dose for an OAR is approximately half the treatment dose.^16^ This is likely to be the case here as single fraction SRS treatments are being considered with an optimal optic nerve dose constraint of 8 Gy.^17^ Therefore, the systematic error component is included in the PRV calculation but the random error component is neglected:


(2) $$m_{\mathrm{PRV}}=1.3\Sigma .\;$$

The guidance in the BIR working party report on calculating CTV to PTV margins through determining the geometric uncertainties in radiotherapy treatment^18^ was used to consider the systematic errors likely to affect optic nerve PRV margins. Technical accuracy and deformation errors are likely to be the dominant systematic errors in this case. Other sources of uncertainty are either assumed to be negligible in comparison with the dominant sources, or in the case of rotation is incorporated into the deformation measurement method.

The technical accuracy is the overall accuracy of the system in delivering dose to a target.^18^ The BIR report on geometric uncertainties states it can be calculated using^18^:


(3) $$\Sigma_{x,y,z\;\mathrm{technical}\;\mathrm{accuracy}}=\frac{a_{x,y,z}}{\sqrt{3}},\;$$

where $a_{x,y,z}$ is the targeting error. The end-to-end accuracy of the CyberKnife system is typically measured to be 0.60 mm during routine quality assurance measurements. Therefore, $a_{x,y,z}=0.60$ was used in eqn (3) giving the systematic technical accuracy error contribution to be $\Sigma_{x,y,z\;\mathrm{technical}\;\mathrm{accuracy}}=0.35\;\mathrm{mm}$.

Deformation error is due to the change in shape of a structure contoured on a planning scan.^18^ Two deformation error models were created to calculate PRV margins for 2 scenarios: firstly, where the full range of motion of the optic nerves measured is considered in a worst-case scenario (referred to as “full range”) and secondly, where an assumption is made that a patient will look in the neutral direction for the majority of a treatment (and could if required be coached to do so)—“neutral focus”.

#### Full range model

The patient keeps their eyes in one, randomly chosen direction for the whole of a fraction. The direction is 1/3 likely to be the neutral position, and 1/6 likely to be in each of the displaced positions.

#### Neutral focus model

The patient is asked to keep their eyes in the neutral position, but does not do so perfectly. The direction changes every 5 min in a 60 min treatment, with half of the time spent at the neutral position and 1/8 time at each of the displaced positions.

The full range of motion deformation error model uses the equation for systematic deformation error given in the BIR geometric errors report^18^:


(4) $$\Sigma_{\mathrm{deform}}^{2}=\;\sigma_{\mathrm{deform}}^{2}\;\left(1+\frac{1}{N_{\mathrm{frac}}}\right),$$

where $\Sigma_{\mathrm{deform}}$ is an estimate of the standard deviation of systematic deformation error, $\sigma_{\mathrm{deform}}$ is the standard deviation of the measured deformation error, and $N_{\mathrm{frac}}$ is the number of fractions (1 in this case). The method for calculating $\sigma_{\mathrm{deform}}$ is shown in Table 4. The difference in co-ordinates of position marker A, at the anterior end of the optic nerves, between the first neutral position scan and the other eye positions was calculated for each optic nerve. These values are shown for an example volunteer in Table 4a. The standard deviation in the difference between each co-ordinate axis, *x*, *y*, and *z*, was then found for each optic nerve. Twice the weighting is applied to the neutral position by the inclusion of the initial neutral zero position and the 2nd neutral scan position in the standard deviation calculation. The pooled standard deviation for each co-ordinate axis was calculated by taking the root mean square of the individual standard deviations, giving the $\sigma_{\mathrm{deform}}\;$values in Table 4b. Putting these values in to eqn (4) gives $\Sigma_{x,\;\mathrm{deform}}=2.28\;\mathrm{mm}$, $\Sigma_{y,\;\mathrm{deform}}=1.70\;\mathrm{mm}$, and $\Sigma_{z,\;\mathrm{deform}}=0.90\;\mathrm{mm}$.

**Table 4.** Displacement from the first neutral scan for an example volunteer and the corresponding standard deviation of deformation error in each co-ordinate axis (a) and the margin calculation for full range model using the entire data set (b).

	Displacement from first neutral scan (mm)								
	Left optic nerve					Right optic nerve			
	*x*		*y*	*z*		*x*	*y*		*z*
(a)									
Neutral	0		0	0		0	0		0
Up	0.6		−1.0	−0.7		0.3	−1.0		0.0
Down	0.2		2.0	0.0		0.7	2.0		0.1
Neutral	0.4		0.0	−0.1		0.0	0.0		0.1
Left	−2.0		0.0	0.8		−2.1	0.0		−0.3
Right	3.0		−1.0	−0.9		3.1	−1.0		1.3
$\sigma_{\mathrm{deform},\;\mathrm{patient}\;n}$	1.60		1.10	0.60		1.67	1.10		0.56
(b)									
$\sigma_{\mathrm{deform}}\;$ (average RMS)		1.61			1.20			0.64	
$\Sigma_{\mathrm{deform}}$ (using eqn. (4))		2.28			1.70			0.90	
$\Sigma_{\mathrm{technical}\;\mathrm{accuracy}}$ (using eqn (3))		0.35			0.35			0.35	
Σ (addition in quadrature)		2.31			1.74			0.97	
$m_{\mathrm{PRV}}$ (using eqn (2))		3.00			2.26			1.26	

The deformation error contribution to the total systematic error in the neutral focus model was calculated using an adapted form of eqn (4):


(5) $$\Sigma_{\mathrm{deform},\;\mathrm{neutral}}^{2}=\;\sigma_{\mathrm{deform},\;\mathrm{neutral}}^{2}\;\left(1+\frac{1}{N_{\mathrm{frac}}}\right)\;+\;\frac{1}{12}\;\sigma_{\mathrm{deform},\;\mathrm{random}}^{2},$$

where $\sigma_{\mathrm{deform},\;\mathrm{neutral}}$ is the pooled standard deviation in difference in position of marker A between the 2 neutral position scans from the same session and $\sigma_{\mathrm{deform},\;\mathrm{random}}$ is the pooled standard deviation in the difference in position of marker A between the first neutral position scan and all positions measured, with 4 times the weighting given to the difference in the 2 neutral positions. The $\frac{1}{12}$ term represents the 12 5-minute periods in the model’s assumptions. This term is similar to the $1+\frac{1}{N_{\mathrm{frac}}}$ term which has the effect of averaging out deformation errors over multiple fractions.

Random error would result in the averaging out of dose distribution but this is unlikely to affect optic nerve doses in this case where the tolerance dose is approximately half of the treatment dose.^16^ However, intra-fraction motion will in practice lead to a non-zero mean displacement of the optic nerves. The impact of this is modelled using the $\frac{1}{12}\;\sigma_{\mathrm{deform},\;\mathrm{random}}^{2}$ term. Here, an assumption is made that over the course of long fraction, optic nerve motion results in a blurring of the effects of deformation. This is analogous to the effect of the $1+\frac{1}{N_{\mathrm{frac}}}$ term over a larger number of fractions. Therefore, the total systematic deformation error in the neutral focus model incorporates deformation due to random error over the course of a long fraction and the difference in position between a planning scan and at treatment, giving $\Sigma_{x,\;\mathrm{deform},\;\mathrm{neutral}}=0.94\;\mathrm{mm}$, $\Sigma_{y,\;\mathrm{deform},\;\mathrm{neutral}}=0.63\;\mathrm{mm}$, and $\Sigma_{z,\;\mathrm{deform},\;\mathrm{neutral}}=0.30\;\mathrm{mm}$.

Adding $\Sigma_{\mathrm{deform}}$ and $\Sigma_{\mathrm{technical}\;\mathrm{accuracy}}$ in quadrature gives the total systematic error standard deviation, Σ. Using this value, eqn (2) was used to calculate an non-isotropic PRV margin for the full range model of $m_{L/R,\mathrm{PRV}}=3mm$, $m_{\mathrm{Sup}/\mathrm{Inf},\mathrm{PRV}}=2\;\mathrm{mm}$, and $m_{\mathrm{Ant}/\mathrm{Post},\mathrm{PRV}}=1\;\mathrm{mm}$ (see Table 4b) and an isotropic PRV margin for the neutral focus case of $m_{\mathrm{PRV}}=1\;\mathrm{mm}$. These margins have been rounded to the nearest millimetre.

### Applying PRV margins to historical plans

**Figure 6.**
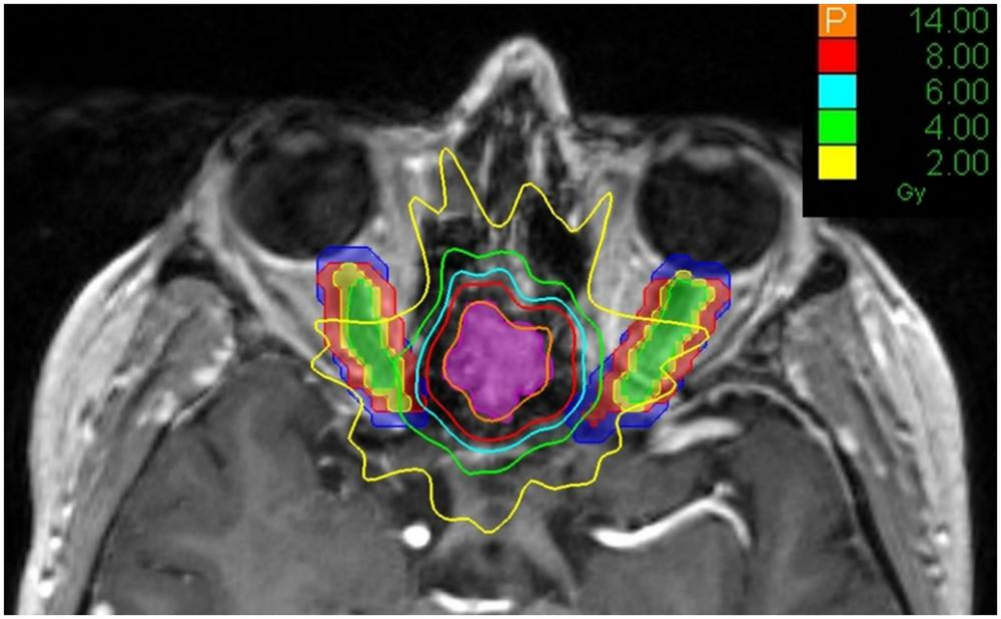
A historical single fraction sphenoid wing meningioma CyberKnife plan (prescription dose of 14 Gy) with the original optic nerve contours in green. These optic nerve contours were grown to PRVs using the following margins: 1 mm isotropic (yellow), non-isotropic (red) and 3 mm isotropic (blue). The non-isotropic PRV has been grown 3 mm L/R, 2 mm sup/inf, and 1 mm ant/post. The optic nerve contour and PRVs are shown in colour wash here and the isodose lines can be seen crossing into these PRV margins.

The dosimetric impact of optic nerve motion was assessed by applying the PRV margins to 8 historical single fraction sphenoid wing meningioma plans (eg Figure 6). These plans were calculated using a 1 mm^3^ dose grid. The dosimetric impact of a 3 mm isotropic margin expansion was also assessed.

The range and mean in maximum dose to 0.03 cm^3^, *D*_0.03 cc_, of the original optic nerve contours and optic nerve PRV margins for these 8 sphenoid wing meningioma plans is given in Table 5. A recent review of tolerance doses for use in SRS and stereotactic ablative radiotherapy (SABR)^17^ recommends an optimal single fraction tolerance dose of *D*_0.03 cc_ < 8 Gy for the optic nerves. An optimal dose constraint of 8 Gy is used here. However, in practice adverse events are likely to be very low up to 12 Gy.^19^ The mean *D*_0.03 cc_ to the optic nerve PRVs increase as the margins get larger but remains within tolerance. Table 5 shows *D*_0.03 cc_ to the original optic nerve contours is within 8 Gy for all plans. This is also true for the 1 mm isotropic PRV, suggesting these plans would have been robust to some optic nerve movement provided the patient kept a neutral focus for the majority of treatment. *D*_0.03 cc_ is above 8 Gy for some of the plans when applying the non-isotropic full range PRV and 3 mm isotropic PRV. This suggests that optic nerve tolerances may be exceeded in cases where patients hold their gaze outside of a neutral position, potentially putting the optic nerves at risk of RION.

**Table 5.** Point (0.03 cm^3^) dose to optic nerves and generated PRV margins for 8 historical sphenoid wing meningioma CyberKnife treatment plans at QEHB.

	*D*_0.03cc_ (Gray) [tolerance dose *D*_0.03cc_ < 8 Gy]	
	Range	Mean
Original contour	1.37-5.04	3.25
1 mm isotropic—“neutral focus”	1.41-6.20	3.68
Non isotropic (3 mm L/R, 2 mm sup/inf, 1 mm ant/post)	1.46-11.67	4.87
3 mm isotropic	1.47-13.39	5.70

## Discussion

In order not to compromise the dose to the lesion and protect treatment outcomes, patient coaching combined with a suitable margin achieves a pragmatic balance between treatment intent and minimization of risk. As expected, maximum optic nerve motion has been measured nearest to the globe and positional variation near to the optic canal is minimal. A 1 mm isotropic margin accounts for any random optic nerve motion when patients are looking in the neutral position for most of the treatment time. Coaching patients to look in a neutral direction during planning scans and treatment, in addition to the use of a 1 mm isotropic optic nerve PRV margin is the most practical solution as it suitably minimizes the risk to the optics whilst maintaining PTV coverage.

The average optic nerve displacement across 5 volunteers measured at the anterior end of the optic nerves by Qing *et al* was 10.8 ± 2.2 mm horizontally and 9.3 ± 0.8 mm vertically.^1^ As expected, the motion measured in this study is smaller compared to previous studies as the eye position protocol restricts eye motion in an attempt to measure non-strenuous eye motion. Even with 1 mm axial slices, there is uncertainty in determining the midpoint of the optic nerves in the axial plane, this increases the uncertainty in the anteroposterior displacement in comparison to the lateral measurements.

Clinically at our centre, CT planning scans are acquired on a flat couch and planning MRI scans are not due to the head coil, resulting in a small difference in the pitch of the head. The vector of the optic nerve motion path may change with patient posture. CT and MRI scans are fused and both guide optic nerve delineation. Treatment on the CyberKnife system is on a flat couch and the system tracks the position of the skull to account for any change in position or rotation. However, soft tissue such as the optic nerves can move independently of the skull. Zappala *et al*^20^ have investigated the effect of head orientation in relation to brain position but the effect of head orientation in relation to optic nerve motion has not been considered in this study.

Our study is limited by the size of our healthy volunteer pool; the reliability of the PRV margins calculated for this project would be improved by increasing the sample size. Another source of uncertainty in the PRV calculation is the modelling of optic nerve motion. An assumption is being made here that optic nerve motion is normally distributed with the peak of this distribution centred on the neutral position. It is not possible to verify this on treatment. Optic nerve motion may not be random if, eg patients are following the CyberKnife robotic arm with their eyes during treatment or are focusing on another object such as the cameras on the ceiling in the treatment room during treatment. These effects can be mitigated with appropriate coaching and education of the patient. Our sample size did not allow us to differentiate between volunteer demographics: there may be a difference in the extent in optic nerve motion between younger and older patients or patients who wear glasses may naturally have a narrower field of view that they consider to be comfortable as glasses do not cover the peripheral vision.

The van Herk model assumes random errors in radiotherapy blur out dose distribution over a large number of fractions. This averaging out of dose distribution due to random errors cannot take place in SRS due to the small number of fractions. The paper by McKenzie *et al*^16^ on calculating PRV margins states that random error can be neglected when an OAR tolerance dose is approximately half the treatment dose, as in this case. However, this article was written for conformal radiotherapy treatments. The validity of applying this assumption to a modern highly precise image guided SRS treatment delivered in 1 fraction requires further investigation. Work has been carried out looking at accounting for the small number of fractions in SRS and SABR compared to conventional radiotherapy for PTV margin calculations^18^ but there is little work on this for PRV margin calculations.

There is increasing interest in the use of hypofractionated SRS for the treatment of optic nerve sheath meningiomas (ONSM). A recent publication by Koç *et al* showed hypofractionated SRS using CyberKnife is effective in treating ONSM.^21^ A 2 mm PTV expansion from the clinical target volume was used by this group.^21^ Future work may involve the use of the optic nerve motion data collected for this study in order to calculate a more accurate optic nerve PTV margin.

## Conclusion

Optic nerve motion has been measured using high resolution MRI and this data has been used to calculate motion inclusive PRV margins for use in SRS planning. Applying these margins to historical SRS treatment plans has shown optic nerve motion may potentially result in the optic nerves receiving a dose above an optimal tolerance of 8 Gy. Therefore, it may be prudent to use patient coaching and education in addition to a 1 mm PRV margin around the optic nerves when treating lesions in close proximity to the distal aspect of the optic pathway, where motion is greatest.
